# Stem-Cell Therapy: Filling Gaps in Oro-Maxillofacial Region

**DOI:** 10.7759/cureus.47171

**Published:** 2023-10-17

**Authors:** Bhumika K Vaswani, Bhushan P Mundada, Nitin Bhola, Priyanka Paul, Amit Reche, Kajal P Ahuja

**Affiliations:** 1 Public Health Dentistry, Sharad Pawar Dental College and Hospital, Datta Meghe Institute of Higher Education and Research, Wardha, IND; 2 Oral and Maxillofacial Surgery, Sharad Pawar Dental College and Hospital, Datta Meghe Institute of Higher Education and Research, Wardha, IND

**Keywords:** adult stem cell, embryonic stem cell, adipose tissue, bone marrow, stem cells

## Abstract

How do stem cells function? Why should we, as dentists, care about stem cells? How might dental procedures be substituted by stem cells? Are stem cells capable of regenerating a tooth or temporomandibular joint (TMJ)? Although the ability to regenerate a destroyed tissue has been known for a while, research into regenerative medicine and dentistry has made significant strides in molecular biology. A paradigm shift in the therapeutic toolbox for dental and oral diseases is likely to result from a growing understanding of biological concepts in the regeneration of oral/dental tissues along with stem cell research, leading to an intense search for "biological solutions to biological problems."

Among other tissues, orofacial tissues effectively separate stem cells from human tissues. Because they can self-renew and produce different cell types, stem cells offer novel techniques for regenerating damaged tissues and curing illnesses. A number of significant milestone successes have shown their practical applicability, traditional biomaterial-based treatments in regenerative dentistry as therapeutic alternatives that offer regeneration of damaged oral tissues rather than merely "filling the gaps."

In order to use these innovative accomplishments for patient well-being, the ultimate goal of this ground-breaking technology, well-designed clinical studies must be implemented as a crucial next step. The review's objective is to briefly synthesize the literature on stem cells in terms of their traits, subtypes, and uses for dental stem cells. It has been highlighted that stem cell therapy has the ability to treat craniofacial abnormalities and regenerate teeth in the oral and maxillofacial regions.

## Introduction and background

Stem cells are defined as unspecialized, immature cells that have the capability to transform into multiple cells [1]. These specific cells have outstanding capacities for self-regeneration and potency and can specialise in numerous cell kinds [1]. New opportunities for dental tissue regeneration in practice are promised by recent advancements in tissue engineering and stem cells. The use of stem cells for treating cancer, degenerative disorders, and other illnesses, as well as to restore damaged or destroyed tissues, is the focus of several clinical trials that have been launched in the fast-growing field of stem cell therapy. Stem cells differ depending on where they are situated and the type of cells they are capable of generating. Stem cell therapy aims to use human cells cultured *in vitro* for therapeutic purposes. Because of their unique capabilities, they are essential for creating advanced technologies [2].

Two fundamental types of stem cells are multipotent and pluripotent, which can only differentiate into a specific population of cells and can differentiate into any adult body cell, respectively. Depending on the source, they could be adult or embryonic stem cells. It is possible to separate embryonic stem cells from the inner cell mass of embryonal blastocysts [2]. Adult stem cells are present in many tissues, including muscles, peripheral blood, bone marrow, the umbilical cord, adipose tissue, skin, tooth pulp, and organs. In dentistry, the oral cavity primarily contains mesenchymal cells [3]. Future treatments involving adult dental ectomesenchymal stem cells seem promising. Adult dental mesenchymal stem cells (MSCs') ability to restore highly vascularized and perfused soft and hard dental tissues is one of the qualities of interest in the field of dentistry [2,3]. Taking all of the above-mentioned factors into account (including stem cells from the apical papilla (SCAP), dental pulp stem cells (DPSCs), periodontal ligament stem cells (PDLSCs), stem cells from exfoliated deciduous teeth (SHED), and dental follicle progenitor cells (DFPCs) that are crucial for tissue regeneration.

Currently, researchers have discovered significant sources of stem cells in oral tissues, as they are a distinct progenitor of stem cells. In order to develop innovative and successful dental applications, dentists and researchers must further define these cells [3]. Tissue engineering is viewed as the future of dentistry [4,5]. The potential for the creation of novel biological treatments, such as dental pulp capping and novel approaches to the treatment of dental root and periodontal disorders, has been increased by the new understanding of tissue engineering and molecular biology [6]. Facial expression, swallowing, and chewing are sensitive processes involving complex anatomical structures consisting of both soft and hard tissues; replacing the oro-maxillofacial structure is challenging. In dentistry, organs and tissues targeted for regeneration include the salivary gland, the condylar cartilage of the temporomandibular joint (TMJ), the craniofacial skeletal muscles, and the tongue.

Various fundamental and translational experiments involve stem cells as well as many different components of tissue engineering, such as bioactive compounds and scaffolds [7,8]. However, a combination of the predicted stem cell-based technologies and the current material-based technologies is required to accomplish effective oral tissue regeneration. The three-dimensional structures for regenerating various tissues can only be created with the help of stem cells, biomaterials, and growth regulators [9]. Animal models have been used to investigate notions in oral tissue and the regeneration of organs for use in dental practice.

## Review

Search methodology

This review carefully gathered literature on the application of stem cells in dentistry, utilising databases such as PubMed, Scopus, and the Web of Science. "Periodontal ligament stem cells", "dental pulp stem cells", "dental follicle progenitor cells", "stem cells from exfoliated deciduous teeth", and "stem cells from apical papilla." Articles suitable for inclusion focused on stem cell applications in dentistry or literature reviews, but publications focusing on the application of these cells in medical and other disciplines were omitted. The inclusion criteria included relevant books, articles, and reviews. The study selection procedure included screening titles and abstracts, followed by a full-text evaluation of relevant papers. The final group of included research offers a thorough analysis of the evidence that is currently available on the use of stem cells in dentistry. The results were combined and analysed to draw meaningful conclusions. The review confirmed the inclusion of high-quality studies that addressed the study’s objectives by adhering to these selection criteria.

Classification of stem cells

**Figure 1:**
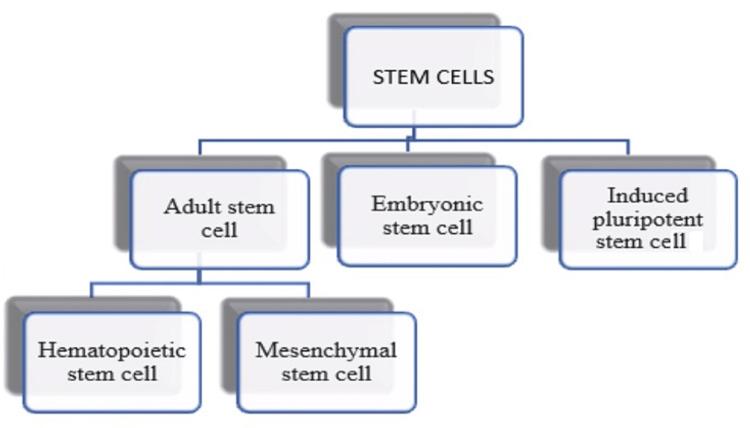
Classification of stem cells The image has been created by the author.

Stem cells are primarily categorized as Embryonic stem cells (ESCs), Adult stem cells, and Induced pluripotent stem (IPS), which exist naturally in humans and have lately been created synthetically by genetic alteration of somatic cells [10,11]. The diagrammatic representation of the classification of stem cells is seen in Figure 1.

Embryonic Stem Cell

Embryonic stem cells are produced by blastocysts, which have 50-150 cells [12]. Cells obtained for the production of embryonic stem cells are cultivated in the undifferentiated inner cell mass of blastocysts, which represents an early stage of embryonic development after fertilization. These are multipotent cells, and the practical viability of these cells is constricted because of ethical considerations. The embryo's inner cell mass is utilized to create embryonic lines [13]. Embryonic stem cells (ESCs) have the capability to divide into three germ layers: endoderm, mesoderm, and ectoderm [14]. Tumorogenesis and immunological rejection frequently occur with ESC [15].

Adult Stem Cell

These are multipotent stem cells [16]. Mature tissues include adult human stem cells (hematopoietic and mesenchymal). These cells can be obtained from different organs, like the liver, tooth pulp, brain tissue, amniotic fluid, pancreas, bone marrow, umbilical cord adipose tissue, and cornea. It stays in a tissue region known as the "stem cell niche." Transplant rejection as well as the production of teratomas are also uncommon in adult stem cells. In addition to humans, other animals can also utilise these cells for cellular repair and organ regeneration.

Induced Pluripotent Stem Cell

Pluripotency is described as the individual potential of a single cell to create all adult creature lineages in response to an embryonic stimulus [17]. Induced pluripotent stem cells (IPS) have three or four stem cell genes and are implanted into donor cells using appropriate vectors. Because of iPS cells' ability to get converted into all tissue, this technique is expected to transform medicine and help the emerging field of "personalised medicine." It utilises an individual's own cells to administer biologically appropriate medications and custom-made therapies.

Sources of stem cell

Treatment of the oral and maxillofacial area can be done with the help of stem cells from many sources such as bone marrow, adipose tissue, and stem cells from the oral and maxillofacial region [17].

*Bone Marrow Stromal* C*ells* *(BMSCs)*

BMSCs are present in adult bone marrow and are peculiar multipotent progenitor cells. The sternum or iliac crest can be used to extract BMSCs. It consists of hematopoietic, mesenchymal, and stem cells. Almost all the structures of the oro-maxillofacial region produce mesenchymal stem cells. Bone marrow has the benefit of having a greater count of stem cells, which allows the development of different types of cells. Isolation of bone marrow cells is possible under general anaesthesia, with discomfort following surgery. Akintoye et al. exhibited important features of iliac and orofacial bone marrow cells from the same person, with orofacial BMSCs having stronger proliferation and osteogenic differentiation potential than iliac crest BMSCs. Furthermore, in an in vivo mouse model, orofacial BMSCs generated more bone, but iliac crest BMSCs produced a large quantity of compact bone that included hematopoietic tissue [18].

Adipose Tissue (ASCs)

Mesenchymal stem cells are abundant in adipose tissue and are intensively researched in the domain of regenerative drugs as a form of stem cells. They can be exfoliated with aspiration after liposuction or lipectomy. ASCs are a form of pluripotent mesenchymal stem cell that may develop into a variety of lineages [19]. ASCs differ from BMSCs in various intrinsic characteristics, but they exhibit robust osteogenesis. ASCs are used as an alternative source of MSCs for bone regeneration therapy. Orofacial bone repair and implant implantation are two of the applications of ASCs [20,21]. Pieri et al. [22] showed in a rabbit model that autologous ASCs transplanted with an inorganic bovine bone scaffold (Bio-Oss1) produced better bone regeneration, implant osseointegration, and vertical bone augmentation, indicating that these cells may be helpful for augmentation of the vertical alveolar bone during implant procedures.

Stem cells from the oral and maxillofacial region

**Figure 2:**
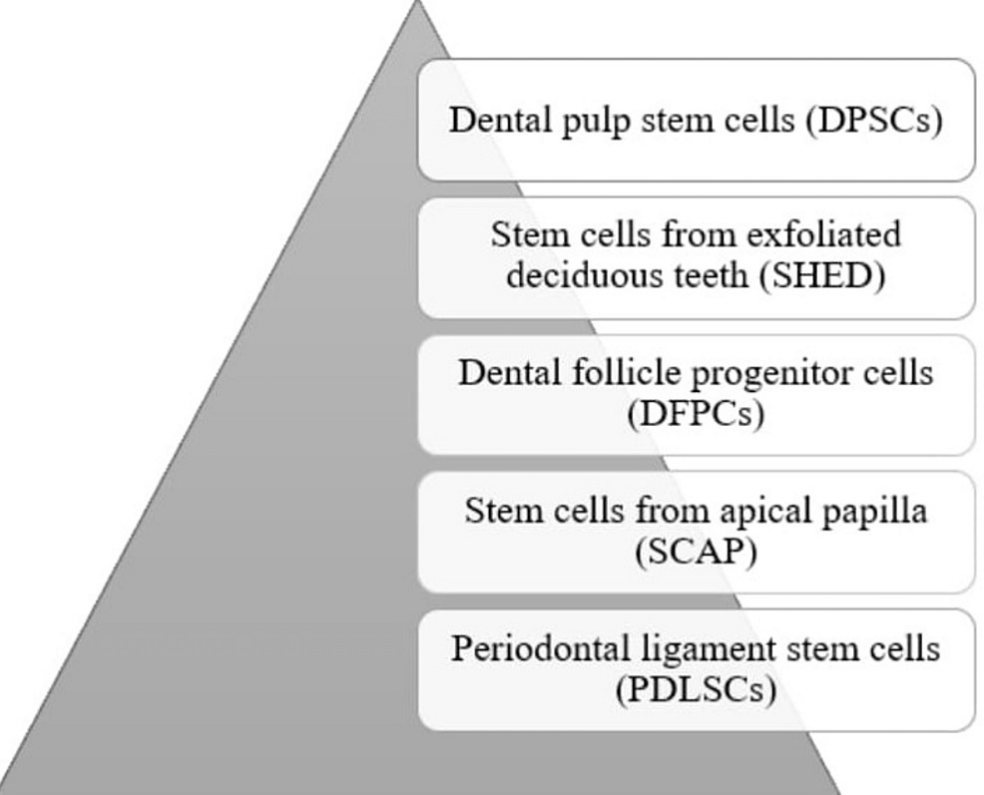
Cells seen in oro facial areas The image has been created by the author.

In oral and maxillofacial areas MSCs are predominant stem cells. Various varieties have been found as well as characterized in oro-facial areas. They are seen in Figure 2.

**Figure 3:**
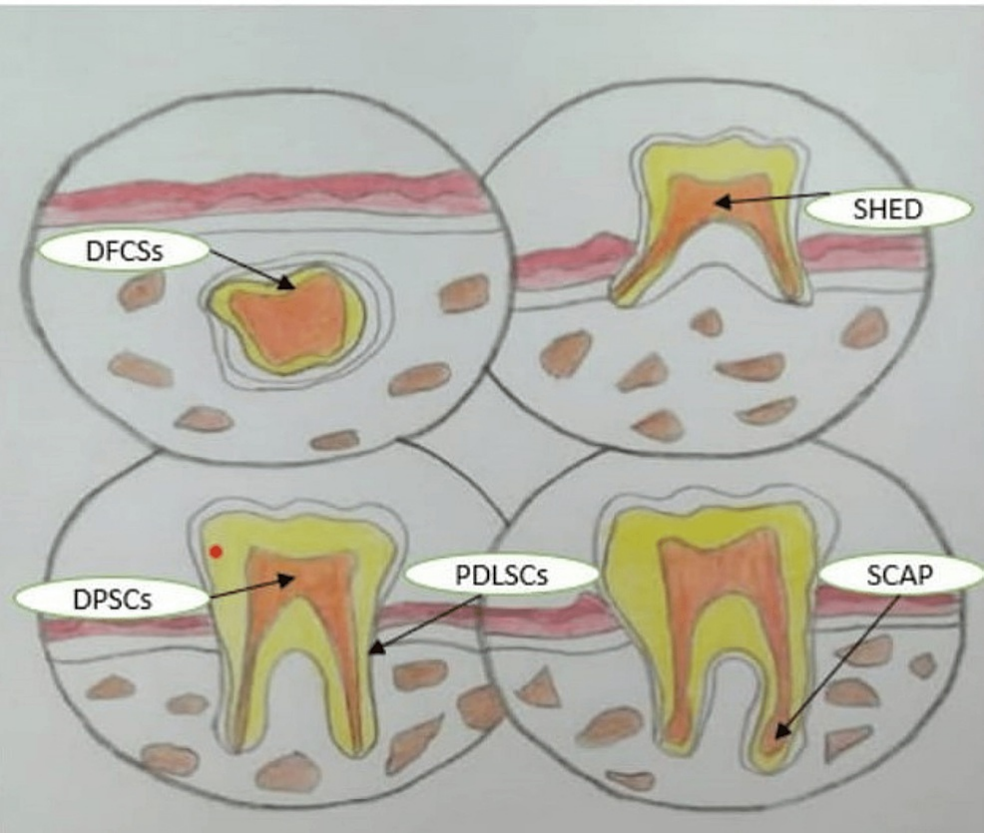
Histopathological representation DPSC- Dental pulp stem cells, SHED- Stem cells from exfoliated deciduous teeth, DFPC- Dental follicle progenitor cells, PDLSC- Periodontal ligament stem cells, SCAP- Stem cells from apical papilla The image has been created by the author.

Histopathological representation of various cells found in oro facial areas is seen in Figure 3.

Dental Pulp Stem Cells (DPSCs)

In 2000, Gronthos et al. extracted DPSCs. In immunocompromised mice, researchers were capable of demonstrating DPSC-derived odontoblast-like cells generating ectopic dentin [23]. Dental stem cells in adult humans were discovered in the dental pulp for the first time in 2000 with phenotypic traits comparable to those of BMSCs [24].

Stem Cells from Exfoliated Deciduous Teeth (SHED)

This is discovered by primary teeth that develop into many cell types, having a higher rate of procreation and more frequent population doublings. Furthermore, by attracting host cells, SHED can specifically trigger the creation of a lamellar bone [25,26]. This unique potential of SHED for bone deposition is illustrated with the help of primary tooth root resorption, followed by bone deposition.

*Dental Follicle Progenitor Cells* *(DFPCs)*

Dental follicle stem cells, which produce PDL cells, cementoblasts, and osteoblasts known as DFPCs, were isolated by Morsczeck et al. [27]. Dental follicles include DFSCs that have the capacity to rebuild periodontal tissues. In the third molar tooth germ at the late bell stage, Ikeda et al. [28] found (TGPCs) tooth germ progenitor cells with significant proliferation activity and capacity to develop in vitro into three germ layers, that is hepatocytes, osteoblasts, and neural cells.

*Stem Cells from Apical Papilla* *(SCAP)*

Sonoyama et al. [29] showed that SCAP, or isolated mesenchymal stem cells from the apical papilla, can produce odontoblast-like cells in vivo. In the papilla tissue at the apex of the roots of growing teeth, stem cells from the apical papilla (SCAP) were discovered [29,30]. SCAP outperforms dental pulp stem cells in immunocompromised mice when it comes to in vitro proliferation and dentin matrix repair. These findings imply that Immature stem cells might be more prominent in "developing" than in "developed" dental tissues.

Periodontal Ligament Stem Cells (PDLSCs)

The periodontal ligament is a source of adult mesenchymal stem cells in dental tissues. Extracted teeth can also be used to retrieve it. Seo et al. extracted PDLSCs from the periodontal ligament of 25 human third molars. They noticed cementoid cells and adipocytes when they were introduced into weak animals [31]. It may serve as a rapid and effective autologous source of stem cells for dental tissue regeneration since they have the capacity to develop into cells that can survive on biocompatible supports [32]. PDLSCs from the alveolar bone surface showed better regeneration when compared to PDLSCs from the root surface, suggesting that PDLSC characteristics may vary depending on where they are taken [33].

Why stem cell research?

Research advancements in stem cells have examined several therapeutic approaches, and the results from pilot-proof concept trials offer a plethora of previously imagined profits. They are helpful in understanding functional genomic investigations, genomic data mining, bioinformatics, and human embryonic gene expression [34]. These can be used to investigate biological processes that aid in the knowledge of human developmental problems like birth abnormalities, malignancies, and so on. It can also provide innovative approaches to developing human illness models for drug discovery and development. It replaces animal toxicology, allowing the medication to reach the market faster [35].

Stem cell storage and transport

In contrast to the decades-long existence of bone marrow and placental cord blood stem cell banks, those that specialise in stem cells collected from teeth are relatively recent. Stem cell-containing tissue samples are to be placed in a screw-top container with a suitable medium that nourishes them while they move. The sample must be sent to the processing storage unit within forty hours [36]. Samples were inoculated and resequenced in the lab to generate clusters of stem cells. By giving appropriate signals to stem cells, the intended cell type may be changed. Most tooth banking services that specify the transfer medium employ balanced salt solutions with the mention of unknown nutrients, such as Hanks buffered saline solution (HBSS) or phosphate-buffered saline (PBS).

Obstacles to overcome for stem cell research

MSCs are found in large numbers in tooth tissues, according to an increasing number of studies. Stem cells, signalling molecules to drive differentiation into appropriate types, such as osteoblasts, cementoblasts, odontoblasts, fibroblasts, etc., and scaffolding material for cellular proliferation support and harvest are required for regeneration of tooth or tooth-related tissues. The difficult problems surrounding factors that affect stem-cell-based bone regeneration are then addressed by the authors. These issues must be resolved for stem-cell-based therapies to be clinically effective. Pre-clinical stem cell research employing MSCs for cell-based immunotherapy and complicated oral tissue and organ engineering are also discussed.

Possible applications of stem cell therapy in the oro-maxillofacial region

Stem cells suited for regenerative purposes have to be subjected to comprehensive regulation of cell control in the body. Only adult MSCs have significant clinical implications in dentistry at the moment. MSC regeneration of periodontium has been extensively researched, with several trials currently reaching the clinic. Effective stem cells must be able to grow into desired tissues or organs, be conveniently extracted, and be processed easily. It should have antimicrobial properties. The pulp, dentin, periodontal ligament, enamel, cementum, craniofacial bones, temporomandibular joint, comprising fibrocartilage, bone, ligaments, tendons, skeletal muscles, subcutaneous soft tissue, and skin, and also including the salivary gland, are all structures of importance to the dentistry profession. During native development, all of these oral, dental, and craniofacial features are generated by mesenchymal cells and/or neural crest-derived cells.

Reconstruction of Dental and Craniofacial Structures

Craniofacial bone grafting therapies include devitalized allogenic and autogenous bone grafting, and osteoconductive biomaterials are also used in craniofacial bone grafting treatments. Donor site morbidity limits autologous bone transplantation, and allogenic bone is frequently lost. These cells possess the ability to help with bone repair and the correction of significant craniofacial abnormalities caused by trauma, enucleation, and tumour resection. Tissue transfer is commonly used to fix a bone defect, although it has limitations such as not being capable of restoring the specific function of the missing area and donor site morbidity, which is followed by scarring, infection, and a lack of function. In cases of severe loss of soft tissues following trauma or surgery, soft tissue repair in the oro-maxillofacial region appears critical. In their experiments, Alhadlaq et al. discovered that when human MSCs are exposed to adipogenic-inducing media, they can transform into adipose cells. Soft tissue reconstruction can be accomplished using adipose cells and an appropriately formed scaffold [36].

Periodontal Regeneration

Clinical research employing material therapies shows that partial periodontal bone loss can be recovered by using bioactive materials, or substances that improve the niche's ability to support resident stem cells in tissue regeneration. Platelet-rich plasma from peripheral blood was combined with autologous mesenchymal stem cells from the iliac crest for periodontal regeneration. After one year, the bone defect had healed significantly and the attachment level had improved. It also demonstrated effective repair and regeneration of the interdental papilla [37].

Regeneration of Damaged Coronal Dentin and Pulp

Novel understandings in tissue engineering, as well as molecular biology, have increased the possibility of the creation of new advanced treatments, for example, pulp capping and many other techniques for the treatment of tooth-related problems [38]. Cells produce an extracellular matrix after differentiation, which is then mineralized. In mouse experiments, bioactive substances in the extracellular matrix were shown to trigger the formation of a dentin bridge or mineralized area in the coronal pulp [39]. The regeneration of pulp tissue requires either the transfer of allogenic, autologous or stem cells or the implantation of the pulp. In their review study, Huang et al. [40] detailed a new approach for endodontically involved immature permanent teeth that required a minimum procedure followed by cleaning with triple antibiotic paste.

Whole Tooth Regeneration

A therapeutic alternative that seemed inconceivable now appears to be a realistic aim. Even now, there are restrictions on replacing lost teeth. Mesenchymal epithelial interactions are required for tooth development. The creation of unique terminal phenotypes, or "helping cells," as a result of signal exchange between these two naive germ layer tissues distinguishes these interactions [41]. Despite the fact that bridges and dentures are much inferior to implants, The absence of a natural structural connection to alveolar bone is the main drawback [42]. They depend on immediate bone integration on the surface of the tooth, which is an unusual interaction in comparison to natural teeth [43].

Proven studies in oral and maxillofacial region

Various studies were evaluated as listed in Table 1.

**Table 1:** Proven studies in the oral and maxillofacial region ADSs- Adipose Tissue, MSC- Mesenchymal stem cells, PRP- Platelet-rich plasma, TMJ- Temporomandibular joint; ECM- Extracellular matrix

STUDY	METHODOLOGY	RESULTS
Grayson et al., 2009 [43]	TMJ was built using tissue engineering. Using digital photographs, condyle-shaped scaffolds were produced from decellularized bone. The scaffold was seeded with stem cells before being placed in a bioreactor chamber which contains culture media.	This method has the potential to overcome a fundamental barrier in providing patient-specific bone grafts for craniofacial and orthopaedic reconstructions: in vitro culture of viable bone grafts of complicated shapes.
Lendeckel et al., 2004 [44]	A child of 7 years of age with a serious head injury had her calvarial defect (120 cm^2^) treated with adipose-derived stem cells. Iliac crest bone transplant and Autologous adipose stem cells were isolated from the gluteal area. Cryoprecipitation was used to create the autologous fibrin glue which keeps the cells together.	This excellent method has raised the prospect of using ADSCs for complex reconstructive surgeries.
Langenbach et al., 2010 [45]	Outgrowth distance from microspheres is significantly reduced when microspheres were treated with an osteoinductive medium for prolonged duration. This decrease was discovered to be related to the incorporation as well as synthesis of ECM proteins.	Murine embryonic stem cells (ESCs) are employed as they are pluripotent and may develop into any form of cell including osteogenic cells. In contrast to cell suspensions or gels, this innovative approach has the extra benefit of allowing for the transplanting of more cells and greater integrity.
Yamada et al., 2006 [46]	For periodontal regeneration, autologous mesenchymal stem cells from the iliac crest were combined with platelet-rich plasma from the peripheral circulation. After one year of follow-up, there was a proper closure of the defect as well as advancement in attachment level, also regeneration of the interdental papilla was seen.	Radiographic examinations revealed that the depth of the bone defect had been minimised. This tissue engineering approach enabled the regeneration of interdental papillae. The use of MSCs in PRP gel is advantageous for periodontal tissue regeneration, treatment of aesthetically sensitive locations, and patient morbidity reduction.

## Conclusions

An entirely new age has begun in the field of dentistry where orofacial bone, parts of teeth, TMJ, and various other regenerations from stem cells extracted from the oral cavity like periodontal ligament, pulp, deciduous teeth, etc., can heal and restore functional and aesthetic harmony in patients. The enormous regenerative potential of embryonic, adult, and induced pluripotent stem cells has been examined, but their use in dental practice is still problematic and constrained by a number of factors that are still out of control, including the high risk of rejection, cell behaviour, the lengthy tooth eruption period, the appropriate crown morphology, and the right colour. Even so, one of the biggest challenges in the dental field for the foreseeable future is the development of biological methods for dental reconstruction using stem cells. Therefore, dental practitioners must be aware of the potential of the developing area of regenerative dentistry as well as the chance of acquiring stem cells while performing standard procedures, which can be saved for later use in autologous therapeutics. In dentistry, stem cell-based treatment is now undergoing intensive research in tissue engineering as well as chair-side cellular grafting techniques, both of which have the potential to produce more predicted regenerative results in the future. Challenges of the study are host-immune response, isolation and identification of appropriate stem cells, long-term stability, and being very costly. Limitations of the use of stem cells are that stem cells in the oral and maxillofacial region are specialized for their specific functions, which may not translate well to other types of tissue repair or regeneration. The number and regenerative capacity of stem cells in this region may decline with age, limiting their effectiveness in older individuals.
